# Prognostic significance of galectin-1 expression in patients with cancer: a meta-analysis

**DOI:** 10.1186/s12935-018-0607-y

**Published:** 2018-08-03

**Authors:** Rongzu Wu, Tingchun Wu, Kai Wang, Shicheng Luo, Zhen Chen, Min Fan, Dong Xue, Hao Lu, Qianfeng Zhuang, Xianlin Xu

**Affiliations:** 1 Department of Urology, XiShan People’s Hospital, 1128 Anzhen Street, Wuxi, 214011 Jiangsu China; 2 Department of Urology, The Third Affiliated Hospital of Soochow University, Changzhou, Jiangsu China; 3 Department of Urology, Sir Run Run Shaw Hospital, The Third Affiliated Hospital of Nanjing Medical University, Jiangning District, Nanjing, Jiangsu China

**Keywords:** Galectin-1, Cancer, Prognosis, Meta-analysis

## Abstract

**Background:**

The prognostic significance of galectin-1 (Gal-1) expression in cancerous patients has been assessed for several years while the results remain controversial. Thus, we performed the first comprehensive meta-analysis to evaluate the prognostic value of Gal-1 expression in cancerous patients.

**Methods:**

We searched Pubmed, Embase and Web of Science to recruit studies on the prognostic impact of Gal-1 expression in cancerous patients. Eighteen studies containing 2674 patients were involved in this meta-analysis until March 30, 2018. Pooled hazard ratios (HRs) with 95% confidence interval (95% CI) were calculated to estimate the effect using random-effects model.

**Results:**

The pooled results revealed that high Gal-1 expression in cancer tissue associated with a poor OS (HR = 1.79, 95% CI 1.54–2.08, P < 0.001). In the subgroup of tumor type, it’s observed that high Gal-1 expression was significant correlated with poor OS in digestive cancers without heterogeneity (HR = 1.94, 95% CI 1.64–2.30, P < 0.001; fixed-effects model; I^2^ = 20.1%, P = 0.276).

**Conclusions:**

Our present meta-analysis indicates that high Gal-1 expression might be a predictive factor of poor prognosis in cancers, particularly in digestive cancers.

## Background

Cancer has been a globally severe health problem. As demonstrated by the data from NCHS, about 1,658,370 people were newly diagnosed with cancers and about 589,430 cancerous patients died in the year of 2015 [1]. Although the survival rate of cancer patients have been increasing in the last decades, the latest diagnostic approaches with better sensitivity and specificity are needed to accurately detecting and treating cancers [2]. Thus, finding better tumor biomarkers is really important to improve the sensitivity and specificity, increasing the efficiency of detecting and treating cancers.

The galectin (Gal) family is a family of endogenous lectins with high affinity for polysaccharides including β-galactosyl residues and a part of animal lectins in the lectin family. Nowadays, 15 members have been found out in the lectin family, which have highly carbohydrate recognition domain (CRD). Galectin-1 (Gal-1) is a secretion from cells and can bind and cross-link glycoconjugates on the cell surfaces, which includes various integrins and glycoproteins of the extracellular matrix (ECM) [3]. Besides, Gal-1 expression is regularly increased in tumor tissues since it can modulate cell adhesion, migration, survival and signaling [4]. At present, it has been clarified by some clinical studies that the expression of Gal-1 has close association with metastasis, recurrence and bad tumor prognosis, which includes cholangiocarcinoma [5], gastric cancer [6–8], gingival squamous cell carcinoma [9], hepatocellular cancer [10–12], renal cell cancer [13], head and neck squamous cell carcinomas [14], ovarian cancer [15, 16], non-small cell lung cancer [17, 18], classic Hodgkin lymphoma [19], laryngeal squamous cell carcinomas [20], glioblastoma [21, 22] and so on. Nevertheless, we still don’t clearly know the impact of Gal-1 on the consistency and magnitude of the prognosis. Therefore, we combined all those published evidences in a systematical manner so as to expose the relationship of Gal-1 and cancerous patients’ prognosis for different kinds of tumors. We attempted to find out whether Gal-1 could help the treatment and prognosis of cancerous patients.

## Materials and methods

This meta-analysis was based on the Preferred Reporting Items for Systematic Reviews and Meta-Analyses (PRISMA) guidelines [23].

### Search strategy

The literature was done via PubMed, Embase and Web of Science databases. Keywords were “carcinoma OR cancer OR neoplasm OR tumor OR tumour” (in all fields) AND “prognostic OR prognosis OR outcome OR survival” (in all fields) AND “Galectin1 OR Galectin-1 OR Gal-1” (in all fields). The latest study was done on March 30, 2018. References of identified literature were also screened to further identify the related researches. Two authors independently searched the database. (Wu Rongzu and Wu Tingchun).

### Criteria for inclusion and exclusion

The following criteria must be met for those literatures eligible for inclusion in this meta-analysis:

1. Gal-1’s expression in cancer tissue.
2. Investigating the association between the level of expression of Gal-1 and survival outcome, which includes overall survival (OS), cancer-specific survival (CSS), disease-free survival (DFS), relapse-free survival (RFS) or progression-free survival (PFS).
3. Offering enough data for the estimation of HR and 95% CI.

When several researches found out the same patient cohort, the whole or the latest cohort was included, with the exclusion of letters, editorials, expert opinions, reviews, case reports and non-human trials. Some researches without critical data for comprehensive analysis were also excluded. Besides, researches with sample sizes less than 40 were not included. The titles and abstracts of determined literatures were independently assessed by two viewers and the irrelevant literature was excluded. The enrolled articles were comprehensively evaluated and further screened by carefully viewing the whole text. Disagreement (if any) was resolved with negotiation.

### Data extraction and quality assessment

Two researchers independently collected the required data from all available studies, including surname of the first author, the date of publication, origin of population, type of tumors, size of sample, mean or median age, gender of patients, stage of tumor, cut-off value, methods for tumor detection, results, and HR and 95% CI of the high Gal-1 expression group versus the low Gal-1 expression group for OS, CSS, DFS, and RFS, if applicable. For those studies without HRs, the survival information was extracted from the raw data (Kaplan–Meier curves) by applying the Engauge Digitizer 4.1, and the data about survival rate was calculated with Tierney’s method [24]. If both the results of univariate and multivariate analysis were reported in a study, only the latter was chosen since its accuracy increased when the confounding factors were considered.

By referring to the Newcastle–Ottawa quality assessment scale (NOS), two reviewers evaluated each study’s quality systematically and independently [25]. A score of 0 was regarded as the poorest quality while 9 the highest quality. A study whose score was no less than six shall be considered as high quality.

### Statistical analysis

The definition of high expression of Gal-1 was made based on the cut-off values given by the authors. The association between Gal-1 expression and cancerous patients’ prognosis was described applying the pooled HRs and their 95% CIs. The evaluation of heterogeneity was made by applying Cochran’s Q test and Higgins I-squared statistics. I^2^ > 50% and/or P < 0.1 suggested a obvious heterogeneity in terms of statistics, according to which a random effect model could be utilized. Alternatively, a model with fixed-effect was needed. If there was the heterogeneity, its source should be explored through the subgroup analysis. The sensitivity analysis was done by omission of each single study so as to evaluate the stability of results. The publication bias was assessed via the Begg and Egger funnel plot. In this meta-analysis, STATA software version 12.0 (Stata Corporation, College Station, TX, USA) was applied. A P-value < 0.05 could suggest statistical significance.

## Results

### Study characteristics

**Fig. 1:**
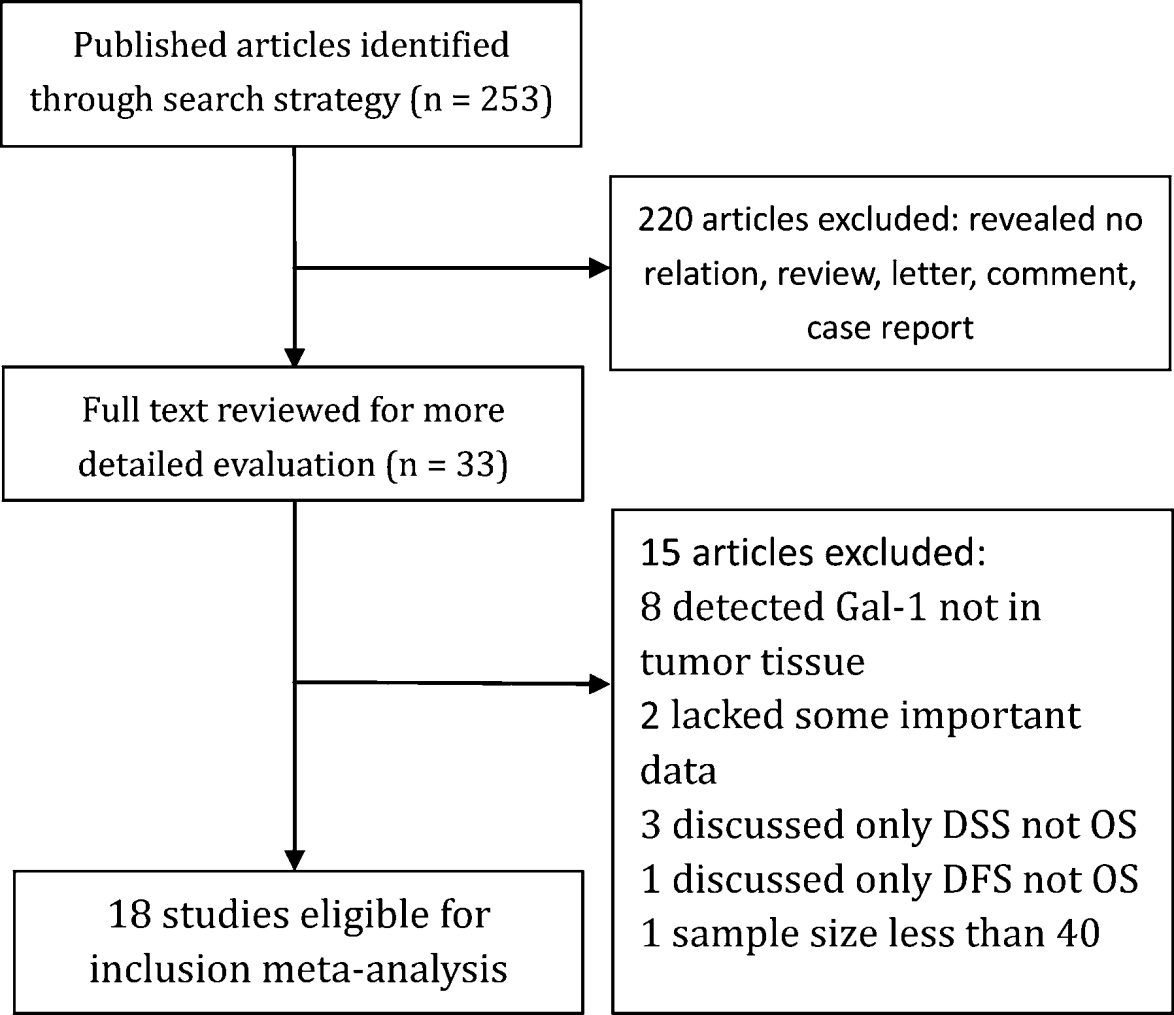
Study identification flowchart

**Fig. 2:**
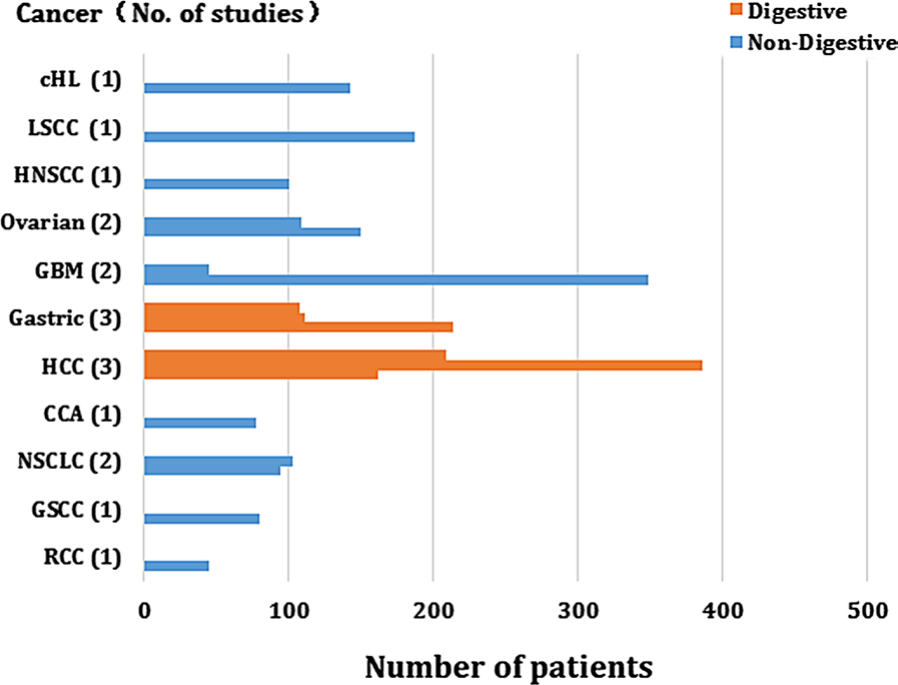
Tumor types are distributed amongst studies and patients. *CCA* cholangiocarcinoma, *GSCC* gingival squamous cell carcinoma, *HCC* hepatocellular carcinoma, *RCC* renal cell carcinoma, *HNSCC* head and neck squamous cell carcinomas, *cHL* classic Hodgkin lymphoma, *LSCC* laryngeal squamous cell carcinomas, *NSCLC* non-small cell lung cancer, *GBM* glioblastoma multiforme

As for the strategy used for search, totally 253 references were retrieved at the beginning. When the titles, abstracts, types of publication and overall text were comprehensively screened, the relationship between Gal-1 expression and the outcomes of patients with various malignant tumors were studied in 33 articles. In addition, 15 articles were not included (Gal-1 was detected not in tumor tissue in 8 articles, some key data were lacked in 2 articles, the sample size of 1 articles was less than 40, Only DSS (not OS) was discussed in 3 articles while DFS (not OS) was discussed in 1 article. Eventually, 18 articles were added into the meta-analysis when the comprehensive assessment was done (Fig. 1). Totally 2674 patients from China, Japan, Hungary, Argentina, Belgium, Germany, Denmark and USA were diagnosed with different cancers, such as cholangiocarcinoma, gastric cancer, gingival squamous cell carcinoma, hepatocellular cancer, renal cell cancer, head and neck squamous cell carcinomas, ovarian cancer, non-small cell lung cancer, classic Hodgkin lymphoma, laryngeal squamous cell carcinomas, glioblastoma and so on. The design of all studies was done retrospectively and the year of publication was between 2005 and 2018. 12 studies targeted Asians while six Caucasians. Totally 18 studies reported OS, while CSS and DFS were assessed only in two studies. OS was selected as the major survival outcome for all of the available studies in our meta-analysis. 7 studies reported HRs with their 95% CIs. Through the graphical survival plots, the data was extracted in 11 studies. The cut-off values of Gal-1 differed in different studies. Table 1 demonstrates the significant features of these 18 available studies. Figure 2 is show how different tumor types are distributed amongst studies and patients.

**Table 1:** Main characteristics of all studies included in the meta-analysis

Study	Country	Cancer	Case number	Median age (year, range)	M/F	Stage	Gal-1 (±) NO.	Cut-off	Multivariate analysis	HR and 95% CI
Wu [5]	Japan	CCA	78	NA	50/28	TNM I–IV	(45/33)	IRS ≥ 3	No	SC
Chen [6]	China	gastric	214	Mean 64.5	129/85	TNM I–IV	(138/76)	IRS ≥ 2	No	SC
Noda [9]	Japan	GSCC	80	Mean 63.8	39/41	TNM I–IV	(22/58)	IHC > 50%	No	SC
Chong [8]	China	Gastric	111	NA	NA	TNM I–IV	(61/50)	IHC > 20%	No	SC
Zhang [10]	China	HCC	209	NA	179/30	TNM I–IV	(128/81)	IHC > 20%	No	SC
Huang [13]	Tainan	RCC	45	NA	31/14	TNM I–IV	(25/20)	H-score > median	No	SC
Le [14]	USA	HNSCC	101	Median 58	84/17	TNM I–IV	(56/44)	IRS ≥ 3	No	SC
Schulz [15]	Germany	Ovarian	150	Median 62 (31–88)	0/150	FIGO I–IV	(102/48)	IRS > 1	No	SC
Chen [7]	China	Gastric	108	NA	61/47	TNM I–IV	(68/40)	IRS ≥ 2	Yes	Report
You [11]	China	HCC	162	NA	127/35	TNM I–IV	(105/57)	IRS ≥ 2	Yes	Report
Wu [12]	China	HCC	386	NA	341/45	TNM I–IV	(189/197)	NA	Yes	Report
Kamper [19]	Denmark	cHL	143	35	78/80	Ann Arbor I–IV	(35/108)	NA	No	Report
Ye [20]	China	LSCC	187	Mean 52.4	179/8	TNM I–IV	(102/85)	NA	No	SC
Szoke [17]	Hungary	NSCLC	94	Mean 58.8	84/10	TNM I–III	(40/54)	NA	No	SC
Carlini [18]	Argentina	NSCLC	103	Median 64 (45–85)	69/34	TNM I–III	(53/47)	IRS > 1	No	SC
Van Woensel [21]	Belgium	GBM	349	NA	NA	NA	(174/175)	Median gene expression	No	Report
Chou [22]	China	GBM	45	NA	27/18	NA	(34/11)	IHC > 35%	No	Report
Chen [16]	China	EOC	109	NA	0/109	FIGO	(91/18)	IRS ≥ 3	Yes	Report

*CCA* cholangiocarcinoma, *GSCC* gingival squamous cell carcinoma, *HCC* hepatocellular carcinoma, *RCC* renal cell carcinoma, *HNSCC* head and neck squamous cell carcinomas, *cHL* classic Hodgkin lymphoma, *LSCC* laryngeal squamous cell carcinomas, *NSCLC* non-small cell lung cancer, *GBM* glioblastoma multiforme, *EOC* epithelial ovarian cancer, *NA* not available, *SC* survival curve, *IRS* immunoreactivity score, *IHC* immunohistochemistry

### Quality assessment

The quality of all those 18 available studies in our meta-analysis was evaluated based on the NOS. The selection bias was observed in each and every study, maybe because only a single type of cancer was included in each study. Therefore, any study in this meta-analysis failed to represent the whole range of cancers. The study quality was between 6 and 7, with a mean value of 6.6. A larger value suggested a better methodology. Thus, the subsequent analysis included all available studies.

### Meta-analysis results

**Fig. 3:**
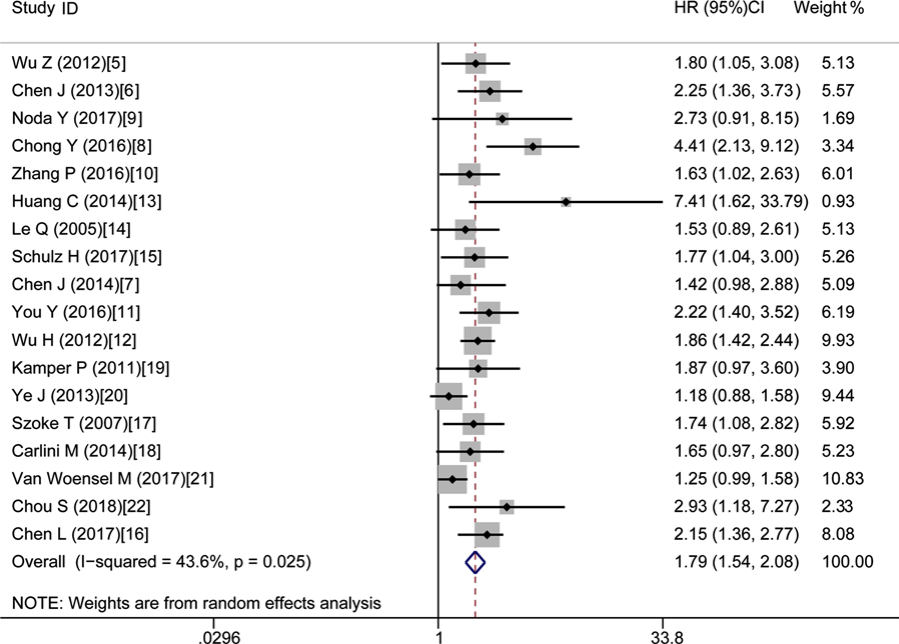
Forest plots of studies assessing HR of high Gal-1 expression in cancers

Table 2 demonstrates the main results of this meta-analysis. Since the studies which evaluated OS have significant statistical heterogeneity (I^2^ = 43.6%, P = 0.025), a model with random-effects was applied to get the HRs pooled. As shown by the statistical results, high expression of Gal-1 is obviously correlated with poor OS in various carcinomas, with the pooled HR of (HR = 1.79, 95% CI 1.54–2.08, P < 0.001) (Fig. 3).

**Table 2:** The pooled associations between Gal-1 expression and the prognosis of cancerous patients (OS)

Outcome subgroup	No. of studies	No. of patients	HR (95% CI)	P value	Model	Heterogeneity	
						*I*^2^ (%)	P
All	18	2674	1.79 (1.54–2.08)	< 0.001	Random	43.6	0.025
Ethnicity							
Asian	12	1734	1.96 (1.60–2.42)	< 0.001	Random	50.5	0.023
Caucasian	6	940	1.42 (1.21–1.66)	< 0.001	Fixed	0.00	0.716
Tumor type							
Digestive system	7	1268	1.94 (1.64–2.30)	< 0.001	Fixed	20.1	0.276
NOT digestive system	11	1406	1.61 (1.33–1.94)	< 0.001	Random	42.5	0.066
Analysis type							
Univariate	18	2674	1.79 (1.54–2.09)	< 0.001	Random	50.0	0.008
Multivariate	4	765	1.93 (1.60–2.32)	< 0.001	Fixed	0.00	0.572
HR obtained method							
Reported in text	7	1302	1.77 (1.42–2.20)	< 0.001	Random	49.2	0.066
Data extrapolated	11	1372	1.77 (1.42–2.20)	< 0.001	Random	47.6	0.039

**Fig. 4:**
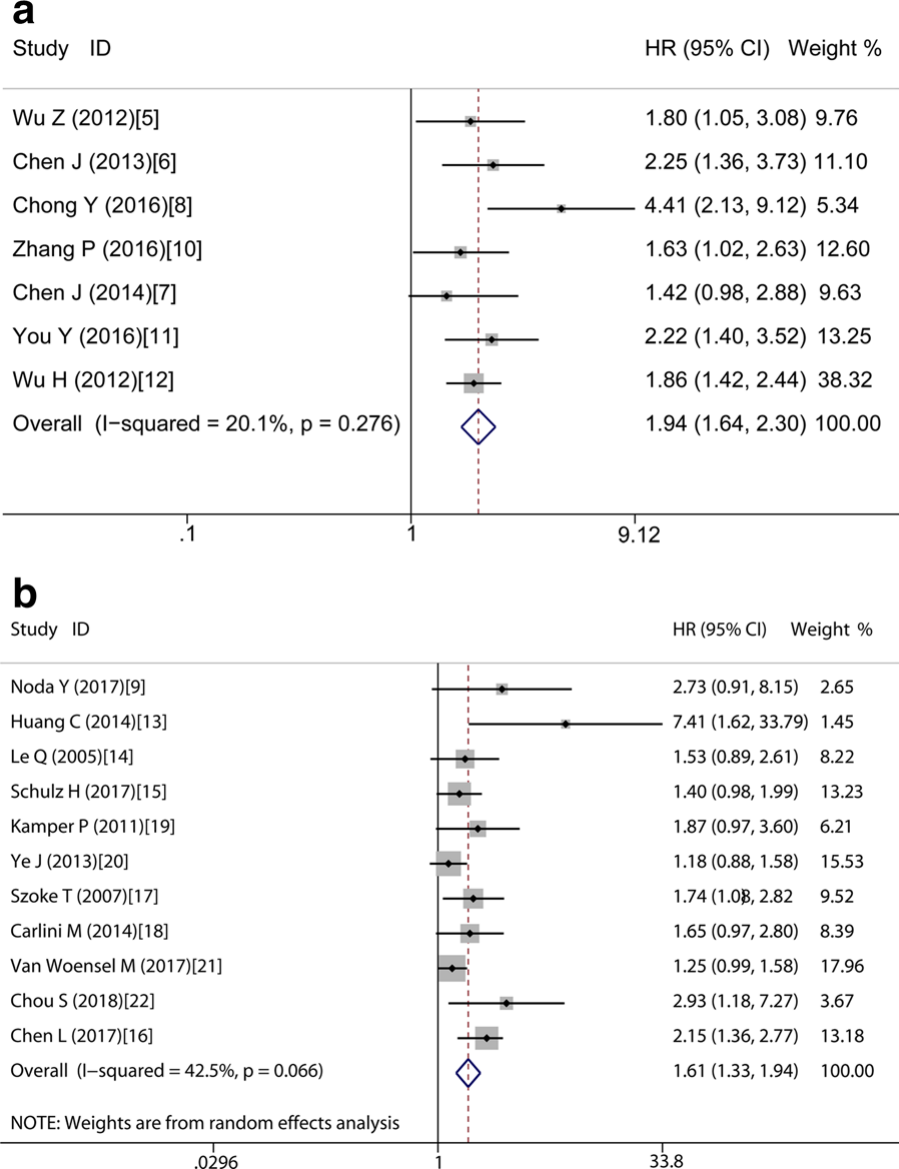
Forest plots of studies assessing HR of high Gal-1 expression in digestive cancers (**a**) and not digestive cancers (**b**)

**Fig. 5:**
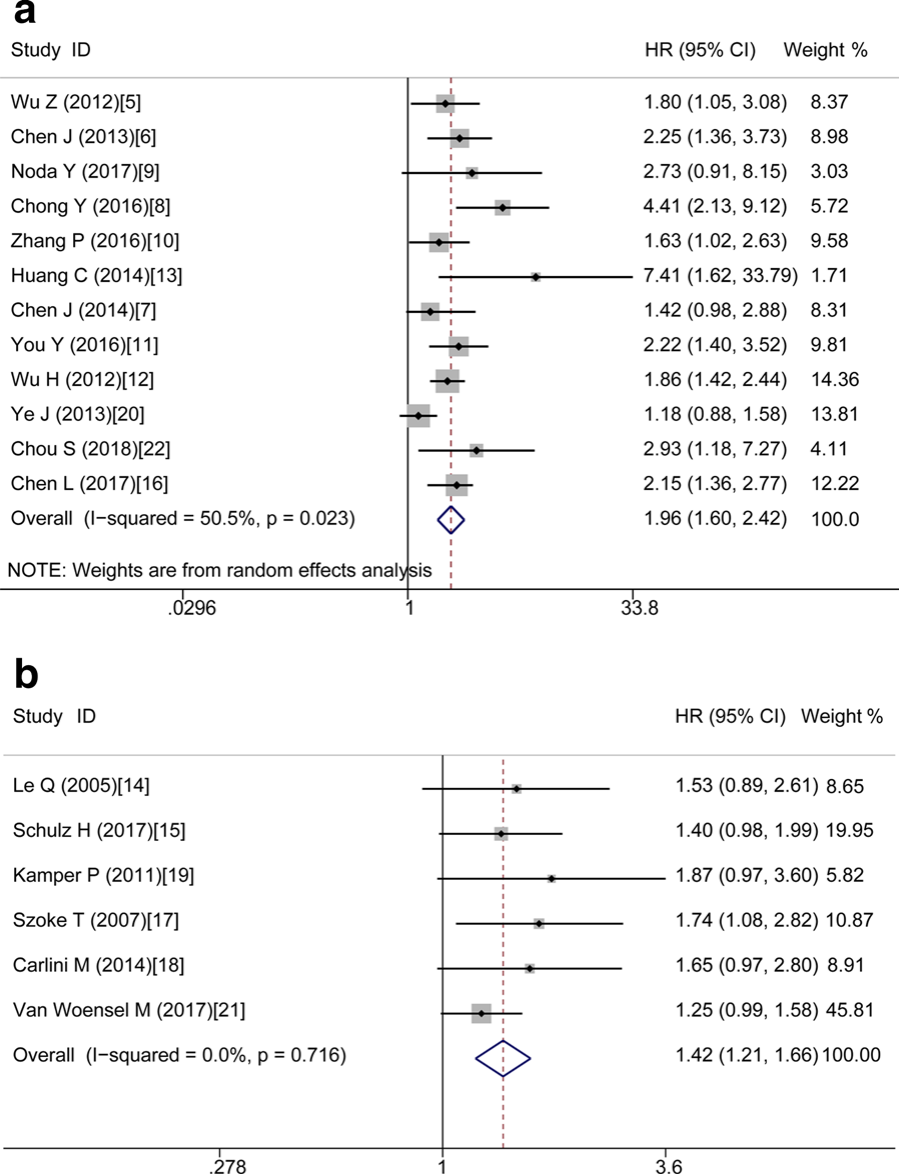
Forest plots of studies assessing HR of high Gal-1 expression in Asian (**a**) and Caucasian (**b**)

In order to study these studies’ heterogeneity, subgroup analysis was done on the basis of four important characteristics, i.e. type of tumor, ethnicity, type of analysis and methods used for obtaining HR. In the subgroup of tumor type, it’s observed that high Gal-1 expression was correlated with poor OS in digestive cancers without heterogeneity (HR = 1.94, 95% CI 1.64–2.30, P < 0.001; fixed-effects model; I^2^ = 20.1%, P = 0.276) (Fig. 4a) and in not digestive cancers with obvious heterogeneity (HR = 1.61, 95% CI 1.33–1.94, P < 0.001; random-effects model; I^2^ = 42.5%, P = 0.066) (Fig. 4b). In the subgroup of Caucasian, there is also without heterogeneity, with the pooled HR of (HR = 1.42, 95% CI 1.21–1.66, P < 0.001; fixed-effects model; I^2^ = 0.00%, P = 0.716) (Fig. 5b). However, in other subgroups, the correlation between high Gal-1 expression and poor OS have statistical significance but with obvious statistical heterogeneity, including Asians (HR = 1.96, 95% CI 1.60–2.42, P < 0.001; model with random-effects; I^2^ = 50.5%, P = 0.023) (Fig. 5a), data extrapolated (HR = 1.77, 95% CI 1.42–2.20, P < 0.001; random-effects model, I^2^ = 47.6%, P = 0.039), reported in text (HR = 1.77, 95% CI 1.42–2.20, P < 0.001; random-effects model; I^2^ = 49.2%, P = 0.066), univariate analysis (HR = 1.79, 95% CI 1.54–2.09, P < 0.001; random-effects model; I^2^ = 50.0%, P = 0.008), Only multivariate analysis with no heterogeneity (HR = 1.93, 95% CI 1.60–2.32, P < 0.001; fixed-effects model; I^2^ = 0.0%, P = 0.572) (Table 2).

### Sensitivity analysis

**Fig. 6:**
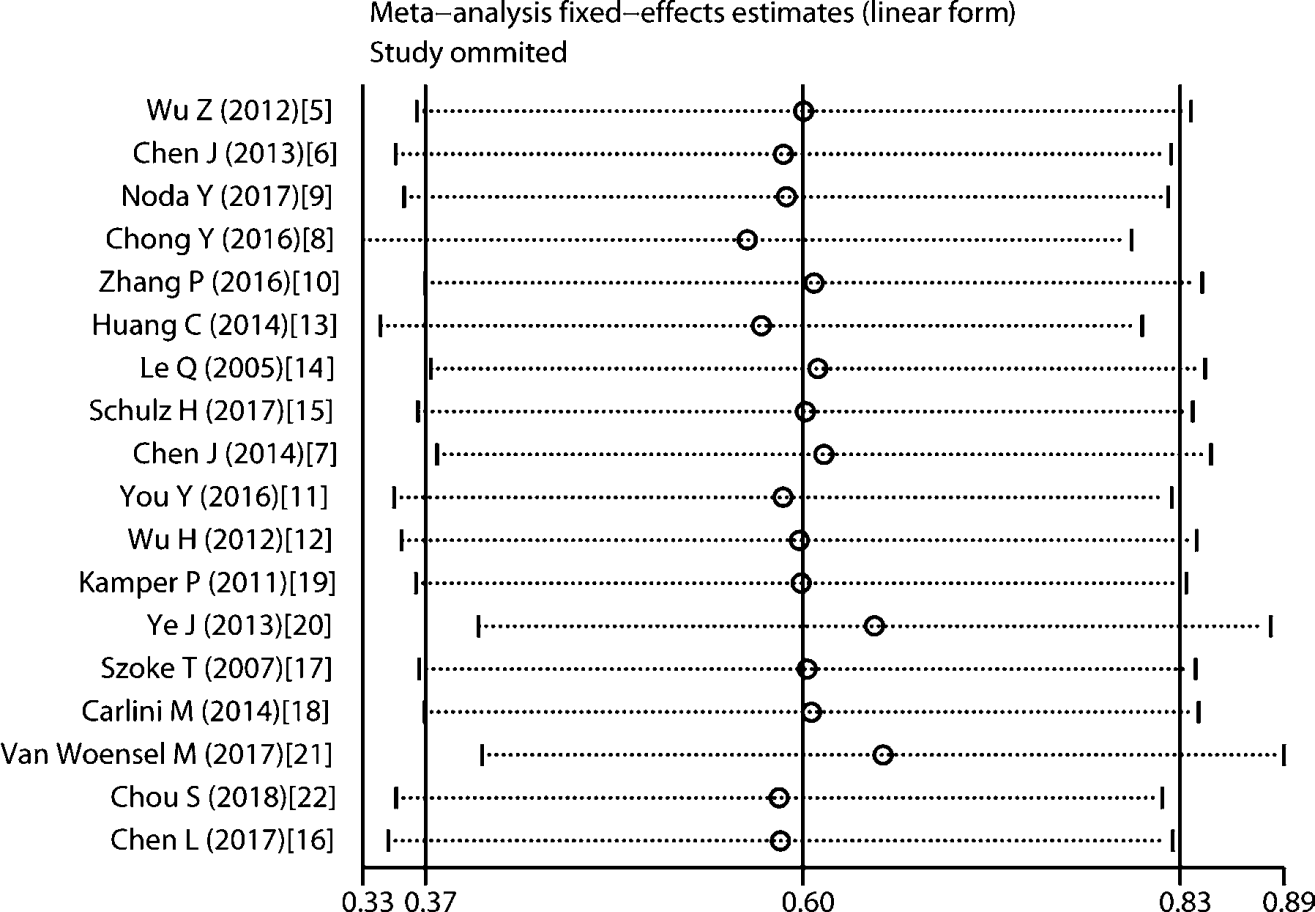
Sensitivity analysis for meta-analysis Gal-1

Sensitivity analysis was done through the sequential omission of single studies using a model with fixed-effects, and the result pattern was not obviously impacted by any single study (Fig. 6).

### Publication bias

**Fig. 7:**
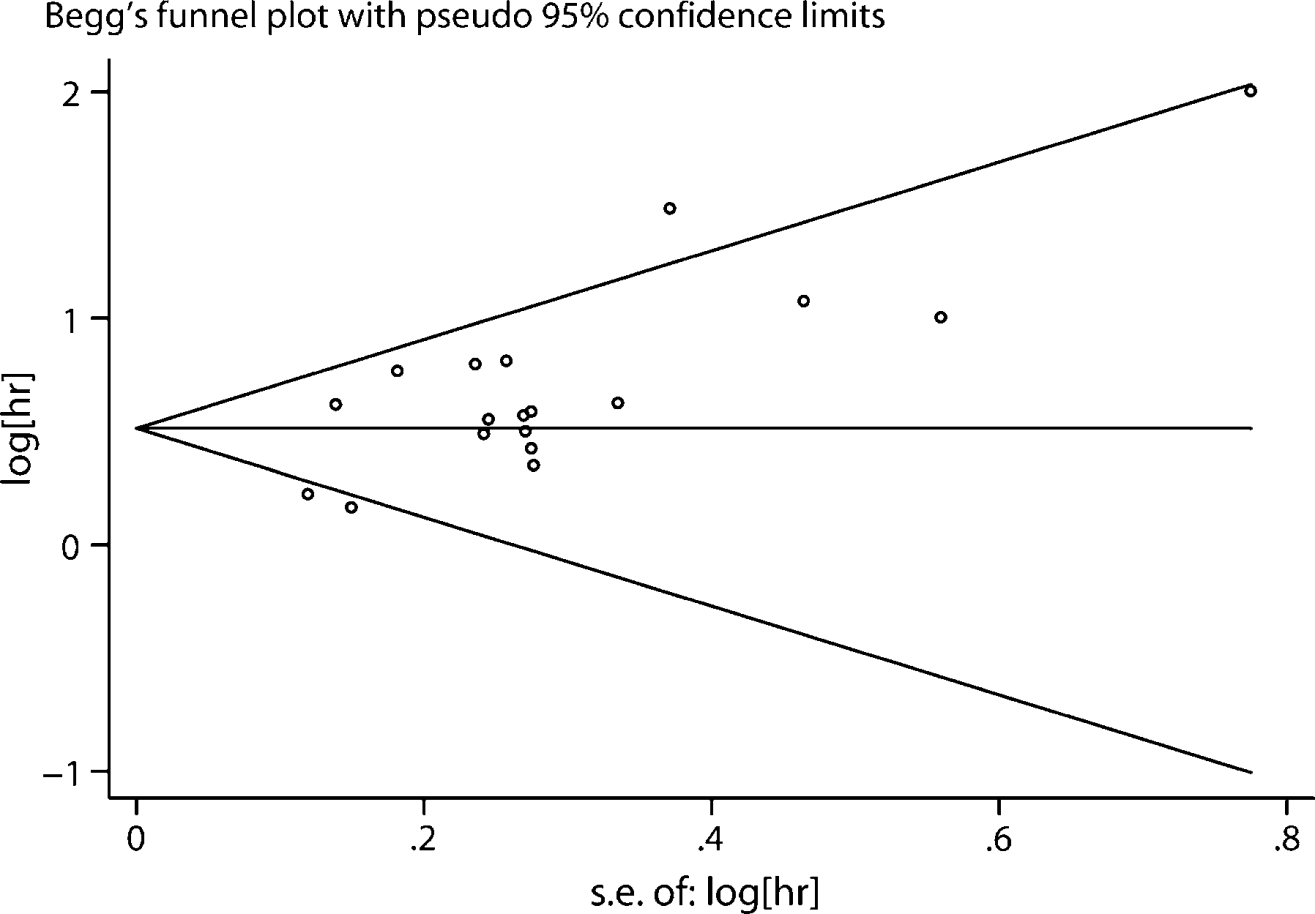
Funnel plots for the evaluation of potential publication bias

The assessment of the publication bias for OS was done through Begg’s funnel plot and Egger’s test. The shape of the funnel plot revealed some evidence of asymmetry (OS, P = 0.103 for the Begg’s test, P = 0.002 for the Egger’s test) (Fig. 7). After adjustment with the trim-and-fill method, the pooled association between Gal-1 expression and OS in tumor patients was also significant (fixed-effects model: HR = 1.49, 95% CI 1.36–1.64, P < 0.001; random model: HR = 1.53, 95% CI 1.30–1.80, P < 0.001), and with significant heterogeneity (P < 0.001). Thus, the results of this meta-analysis are reliable.

## Discussion

Although the past decades have witnessed great achievements in preventing and treating cancers, lots of cancers can’t be treated or cured. Two of the major reasons are the lack of effective biomarkers required for early detection and the inefficient treatment of cancers diagnosed at the terminal stages. As shown by many researches, the expression of Gal-1 has statistically clinical significance, indicating Gal-1 might be a potential biomarker for the prognosis of cancers. Gal-1 is the prototype member of the Galectin superfamily, with the characteristics of high affinity binding to *β*-galactosides via a well-conserved carbohydrate recognition domain (CRD) [26]. Gal-1 can bind and cross-link glycoconjugates on the cell surfaces and regulate various biological processes, such as T cell homeostasis, resolution of inflammatory responses, host–pathogen interactions, selective deletion of specific thymocytes during T cell development, fetomaternal tolerance, and embryogenesis [3, 27–29]. Besides, it’s known that high levels of Gal-1 expressed broadly over primary tumor sections via immunohistochemistry [30–32]. In the tumor microenvironment, Gal-1’s upregulation benefits the tumor growth and reinforces the tumor progression by the modulation of cell motility [33], inducing apoptosis of activated T cells [34], mediation of cell adhesion [35], and participation in tumor angiogenesis [36]. Besides, intracellular Gal-1 links oncogenic H-Ras to promote its anchorage to plasma membrane and stimulate the extracellular signal-regulated kinase (ERK) signaling pathway for neoplastic transformation [37]. Indeed, in most of the clinical studies, it’s reported the raised level of Gal-1 is connected to the poor prognosis [7, 11, 13, 20, 22]. On the other hand, although the relationship between Gal-1 expression and tumorigenesis has been studied intensively, no comprehensive analysis is done for the available data. Therefore, the consistency and scope regarding Gal-1’s prognostic impact are unknown. As far as we know, except this one, there is no other meta-analysis focusing on the association between Gal-1 expression and cancerous patients’ survival rate.

This study demonstrates the relationship between high expression of Gal-1 in cancer tissue and a poor OS in cancerous patients with obvious statistical heterogeneity (HR = 1.79, 95% CI 1.54–2.08, P < 0.001; I^2^ = 43.6%, P = 0.025). Nevertheless, in the analysis of subgroup, the elevated galectin-1 expression was considered as a bad prognostic marker in cancerous patients for OS, regardless of the kind of tumor, ethnicity, the kind of analysis and the method of obtaining HR. In particular, no obvious statistical heterogeneity is observed in digestive cancers, Caucasian and multivariate analysis (I^2^ < 50%, P > 0.1). Thus, we believe that the heterogeneity of this meta-analysis mainly due to the difference in tumor type, patient, and type of analysis. In addition, all cut-off values are reported in the study, which may also lead to heterogeneity due to the absence of uniform standards. In summary, Gal-1 might function as a poor prognostic biomarker for cancerous patients, in particular, those of digestive origin and Caucasian.

This study is limited on several aspects. First, because of the missing of a unified cut-off value in Gal-1 expression, various cut-off values are utilized. This possibly exerts influences on the validity of Gal-1 as a predictive marker in the prognosis of cancer. Second, some HRs were computed according to the data gained from the survival curves, which unavoidably contributes to minor statistical errors. Finally, significant heterogeneity was shown, possibly because of the differences in patient origin, date of publication, kind of tumor, tumor stage, method used in the experiment, follow-up time, cut-off values and others. Since the current analysis has some limitations, more excellently-designed large-sized researches including more kinds of tumor should be done in the future.

## Conclusions

This meta-analysis combined all researches, and attempted to study the relationship between the high expression of Gal-1 and the survival rate of cancerous patients. High Gal-1 expression can be used as a poor prognostic marker for tumors. This conclusion should be regarded carefully since the current analysis has some limitations. Given the sparse data, additional studies regarding Gal-1 are warranted.

## Abbreviations

HR: hazard ratio
95% CI: 95% confidence interval
CCA: cholangiocarcinoma
GSCC: gingival squamous cell carcinoma
HCC: hepatocellular carcinoma
RCC: renal cell carcinoma
HNSCC: head and neck squamous cell carcinomas
cHL: classic Hodgkin lymphoma
LSCC: laryngeal squamous cell carcinomas
NSCLC: non-small cell lung cancer
GBM: glioblastoma multiforme
EOC: epithelial ovarian cancer
NA: not available
SC: survival curve
IRS: immunoreactivity score
IHC: immunohistochemistry
OS: overall survival
CSS: cancer-specific survival
DFS: disease-free survival
RFS: relapse-free survival
PFS: progression-free survival
DSS: disease-specific survival
